# Integrated Community-Clinic Model of Optimized Implementation Strategies to Increase Early Detection of Breast and Cervical Cancers in Kenya: Protocol for a Pragmatic Randomized Controlled Trial

**DOI:** 10.2196/87850

**Published:** 2026-05-18

**Authors:** Robai Gakunga, Lilian Mburu, Zipporah Ali, Mark Krudy, Mary Inziani, Daniel Katua, Clara Kerich, Oscar Agoro, Sujha Subramanian

**Affiliations:** 1 Implenomics, 8 The Green, Dover, DE, 19901, United States; 2 Kenya Medical Research Institute, Nairobi, Nairobi County, Kenya; 3 Machakos County, Kenya, Machakos, Kenya; 4 Nakuru County, Nakuru, Kenya; 5 Nyeri County, Nyeri, Kenya

**Keywords:** breast cancer, cervical cancer, screening uptake, randomized controlled trial, implementation science

## Abstract

**Background:**

Kenyan women experience a high burden from breast and cervical cancers. Screening can result in earlier diagnosis, but its uptake is extremely low. The proportion of women up to date with screenings per guidelines is low, implying that interactions between providers and women are reduced.

**Objective:**

We will test complementary strategies in community and clinic settings that can improve community–clinic linkages and address multilevel barriers. We aim to conduct randomized trials to compare a C3Link (cancer community–clinic linkage) program with enhanced usual care. We will (1) conduct a cluster randomized trial to assess the short-term and longer-term impact of the C3Link package of strategies, (2) use a mixed methods approach to assess multilevel implementation outcomes, and (3) perform cost-effectiveness and return on investment analysis.

**Methods:**

We will create the C3Link package aimed at increasing breast and cervical cancer screenings, and adopt a study design to assess and evaluate sustainability in 3 counties: Machakos, Nakuru, and Nyeri. Three noncontiguous communities for each of the 9 study clinics (3 per county) will be randomly assigned to one of the study arms using the R (R Foundation) program. The intensity of the strategies varies from low (awareness campaign for the enhanced usual care arm), to medium (C3Link Core arm with study community health worker–led group education and study community health worker–embedded clinic teams enhanced with practice facilitation), and to high (C3Link Plus arm with C3Link Core and a predetermined sequence of individual strategies). Women aged 30 to 55 years (1 per household) will be eligible to participate and could select a support person for inclusion. Trained data collectors will conduct surveys for women, their support persons, and from providers in the clinics using questionnaires, tracking tools, and focus group discussion guides. We will collect data and conduct interviews and focus groups to identify underlying moderating factors associated with the successful implementation of strategies. We will use implementation science methods to evaluate the effectiveness, implementation outcomes, and cost-effectiveness of the C3Link program compared with the enhanced usual care.

**Results:**

This study was funded in July 2022, received institutional review board approvals in November 2022 (United States) and June 2023 (Kenya). It was registered on ClinicalTrials.gov in September 2024. Enrollment of this study’s cohort began in September 2024 and was concluded in November 2024. We began implementing the interventions in 2025 and will report on our primary end points on screening uptake and follow-up in 2027 based on 24-month follow-up data.

**Conclusions:**

The C3Link Program is expected to yield better screening and follow-up outcomes than the control but will require more resources. Incremental cost-effectiveness of each strategy in the outlined sequence will be determined.

## Introduction

### Background

In Kenya, breast and cervical cancers are the most commonly diagnosed cancers and the leading causes of mortality among women [1]. Most of these cancers are diagnosed at a late stage: 60% of breast cancers and 53% of cervical cancers are diagnosed in stages III or IV [2-5]. In contrast, about two-thirds of breast cancers are identified at an early stage in the United States [6]. Treating late-stage cancers is more challenging, often with a poor prognosis, which results in high levels of premature mortality and morbidity in Kenya [7]. For example, the mortality-to-incidence ratio ([mortality rate/incidence rate]*100), for breast cancer in Kenya is 48% compared to 12.7% in the United States and 14.9% in the United Kingdom [1,8,9]. For cervical cancer, it is 53.9% in Kenya, compared to 34.9% in the United States and 26.7% in the United Kingdom [1,8,9]. Notably, the incidence rate for breast cancer in the United States and the United Kingdom is more than double that in Kenya, and the incidence rate for cervical cancer in Kenya is more than 5 times that in the United States and the United Kingdom [1,8,9]. Mitigation measures in the United Kingdom include human papillomavirus (HPV)–based screening—a high-performance test—and mammography at personalized intervals, hence early detection and interventions for cervical and breast cancer, respectively, and HPV vaccination for cervical cancer for both boys and girls younger than 10 years [10,11].

There is abundant evidence that screening results in an earlier stage of diagnosis and, in fact, that screening can prevent cervical cancer [12-16]. National estimates for Kenya, of women who have ever received screening, are 14% and 48% for breast and cervical cancer, respectively, where the 48% in cervical cancer includes all methods, while HPV testing accounts for less than 6% [5,17]. The proportion of women up-to-date with screenings within intervals recommended by guidelines is likely much lower [18]. This low level of engagement in the screening process also means that health care providers have limited opportunity to educate women to identify cancer symptoms, which can further facilitate early diagnosis. Early detection of cancer can occur by increasing screening uptake and improving knowledge of symptoms.

Breast cancer screening guidelines for women in Kenya previously included mammography from level 4 health facilities (subcounty hospitals to national referral hospitals), and clinical breast examinations (CBE) from level 2 facilities (dispensaries and private clinics) onward, with referrals for screening mammograms to higher levels as appropriate [5]. Upon testing these guidelines, logistical challenges and health system barriers were found to be a limitation; hence, the Kenya Ministry of Health (MOH) now recommends CBE at all levels with referrals to levels 4, 5, and 6 (subcounty hospitals to national referral hospitals) for diagnostic mammography (2024 guidelines), but still acknowledges mammography as the recommended screening method in the average risk population [5]. The World Health Organization and Breast Health Global Initiative support early detection by using CBE because population-wide mammography can be expensive and logistically challenging in low-resource settings. Properly done CBEs can downgrade breast cancer, improve outcomes, and reduce costs of care. The Kenya breast cancer screening guidelines recommend that women with average risk initiate screening from ages 25 years up to age 34 years with CBE every 3 years, combine CBE and ultrasound during 35‐39 years every 2 years, combine CBE and mammography during ages 40‐55 years annually, reduce this frequency to biennially during ages 56‐74 years, then determine continuation based on individual factors. Women at higher risk are to receive intensive screening and/or genetic counseling [5].

The rationale for cervical cancer screening in Kenya is to achieve sufficient coverage based on the WHO’s Cervical Cancer Elimination Strategy’s recommendation (achieve 70% of women screened by a high-performance test (HPV test), aiming to screen every woman aged 30‐49 years nationwide at least twice [17]. HPV testing also has the advantage of self-sample collection at level 1 (community level) of the health system, which can be aided by community health workers (CHWs) and is deemed culturally acceptable, reducing embarrassment and stigma associated with pelvic examinations. Population-wide cervical cancer screening targets women aged 25‐49 years and recommends that women 30 years of age or younger receive Papanicolaou tests and those older receive HPV tests; 5-yearly for those uninfected with HIV and 3-yearly for those infected with HIV [17]. Multistakeholder efforts have seen marked improvement of screened women aged 25‐49 years from 5% in 2018 to 48% in 2024 [5]. Even with this improvement, Kenya still falls short of the 70% WHO target.

Kenya’s Cancer Screening Guidelines recommend CBEs and mammograms for breast cancer screening, and ultrasound when recommended by health care providers, and the National Cervical Cancer Elimination Plan recommends HPV DNA testing for cervical cancer screening [5,17]. To spearhead screening access, the government has invested in screening by installing mammography machines and updated laboratory services in each county. The screening infrastructure is substantially underused even though CBEs for breast and visual inspection with acetic acid/visual inspection with Lugol’s iodine for cervical cancer screenings are offered free of charge at many public health facilities, which eliminates a key barrier related to cost [19]. Our extensive formative research, supported by other published studies, revealed that, although women generally know about screening, they have a limited understanding of the available cancer screening services [20-25]. A key gap is the lack of linkages between women in the community and clinics where services are provided, which perpetuates the situation in which screening services are available, but there is no demand for the services.

At the individual level, women lack knowledge of their risk factors and the role of screening as a prevention and early detection tool [4,21,22]. These factors, along with fear of the tests, limit their personal agency to seek screening [6,7,26]. At the interpersonal level, social norms that perpetuate fatalism, cancer stigma, and gender roles in decision-making also discourage care-seeking behavior [26-29]. At the structural level, barriers include provider knowledge, discomfort discussing sensitive topics [21,30], provider stigma, and inadequate communication of test results. For example, more than one-third of women did not receive their test results in a screening pilot study in Nyeri County [31].

**Figure 1.**
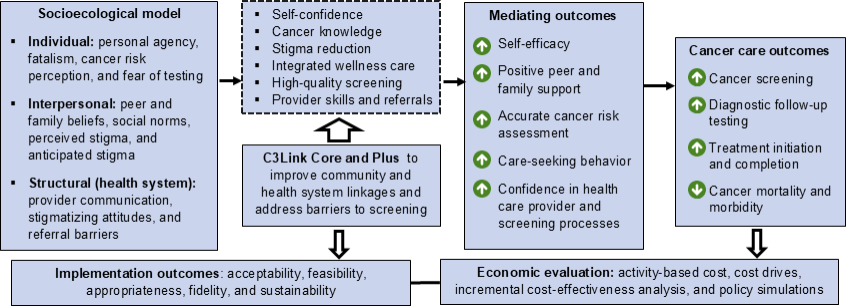
Conceptual framework for creating linkages and addressing barriers to reduce cancer mortality; C3Link study. C3Link: cancer community–clinic linkage.

The theoretical framework used, based on the socioecological model [32], is presented in Figure 1. Drawing on the literature and prior studies already cited, we will create the C3Link (cancer community–clinic linkage) multicomponent strategies targeted at barriers at the individual, interpersonal, and structural levels. These strategies are guided by the COM-B (Capability, Opportunity, and Motivation–Behavior) change model [3], in which capacity (CHW-facilitated cancer knowledge and risk assessment), opportunity (availability of high-quality screenings and confidence in the health care system), and motivation (self-efficacy, peer support, and care-seeking behavior) [4,6,7,12-16,26,33-35] interact to facilitate behavior change. Given the extremely low levels of cancer screening in Kenya and the importance of screening to reduce premature mortality, community stakeholders deemed it unethical to use the “current standard of care” as the comparison arm. We will, therefore, vary the intensity of the strategies from low (awareness campaign for the enhanced standard of care), to medium (C3Link Core with CHW-led group education and CHW-embedded clinic teams enhanced with practice facilitation), and to high (C3Link Plus with C3Link Core and a predetermined sequence of individual strategies). We will test the hypothesis that the C3Link (Core or Plus) combination of CHW-led community strategies for women (individual level) and their families (interpersonal level), along with health system practice facilitation (structural level), will increase cancer screening compared to the enhanced standard of care. Furthermore, we anticipate that CLink Plus will improve screening outcomes the most but will also require the most resources. Assessment of implementation outcomes, using the Proctor framework [36] and incremental cost-effectiveness analysis, will guide the selection of optimal strategies.

### Objectives

This cluster randomized controlled trial aims to assess the short-term and longer-term impact of a package of strategies (C3Link Program), assess multilevel implementation outcomes, and perform cost-effectiveness and return on investment analysis to support scale-up of effective approaches and strategies.

Kenya has expended substantial resources to improve their screening infrastructure, but this investment continues to be underused due to logistical and health system barriers. A key gap, as described in the background section, that perpetuates the low breast and cervical cancer screening rates in Kenya is the lack of community–clinic linkages, anticipated or perceived stigma, and reduced self-efficacy to undergo screening. The planned assessment of the C3Link packages can provide the evidence to overcome barriers and to justify further investment in cancer screening, which is essential to scale up the Kenyan guidelines’ recommended services and inform policies under universal health coverage, which is being adopted across Kenya. Our main objective is to develop and test complementary strategies in community and clinic settings that can improve C3Linkages and address multilevel barriers.

We will create the C3Link package of implementation strategies that will include CHW-led strategies in the community and practice facilitation in the clinic setting.

For the community setting, there is growing evidence on the important role CHWs play in addressing individual and interpersonal barriers to improve knowledge and encourage women to seek medical care [12,13,33,37]. In Kenya, pilot studies in Meru and Nyeri counties have shown that CHWs can facilitate uptake of cancer screening [14,31]. We will use the lessons learned to develop 2 packages of strategies, the C3Link Core and C3Link Plus models. For C3Link Core, CHWs will deliver group education sessions to women and families (male partner or other support person), which is an evidence-based, low-cost strategy to promote cancer screening [15,16,34,35,38]. In our formative research, we found that many women were comfortable discussing issues related to fear and misinformation in individual rather than group settings [19-21]. Therefore, we hypothesize that group education sessions will motivate some women to screen, but others will require an individual-level strategy, or a combination of multiple individual-level strategies [39], to seek screening. To optimize available resources and adopt a tiered process through which only women who require multiple strategies are offered them, C3Link Plus will include the C3Link group education sessions and then add on individual evidence-based strategies [40-42] that increase in resource intensity in a sequential order, targeting women who remain unscreened at 1-month intervals. Women who remain unscreened after group sessions will first receive one-on-one education, followed by motivational interviewing, and, finally, navigation to address specific barriers.

For the clinic setting, the C3Link Core and Plus will use practice facilitation in clinics to foster team-based care with CHWs from the community embedded within the clinic teams to build optimal C3Linkages and communication channels. Practice facilitation is an implementation strategy that has been used successfully in varied settings to build health care workforce capacity and support changes to improve outcomes in health care facilities [43-46]. CHWs serving as team members in the clinic setting can return test results to women and cocreate optimal processes in screening clinics to support completion of follow-up recommendations. We will also provide education to health care providers to improve communication and reduce any stigmatizing behaviors. Furthermore, based on feedback from women in our study communities, we have adopted a holistic women-centered approach (supported by over 80% of the women [47]) to increase uptake of cancer screenings. The community education will include general wellness and disease prevention, and wellness examinations (hypertension and diabetes screening) will be offered during screening clinic visits and annually.

We will supplement the infrastructure investments already in place by providing additional training on cancer screening procedures. Through the practice facilitation process, we will implement a learning collaborative that will offer peer-to-peer consultations to improve screening capacity. In prior discrete choice experiments, which are a quantitative stated preference method to systematically assess attributes women value most in deciding to undergo cancer screening, the competency of the provider was the key attribute [47,48].

The main aim of this study is to conduct randomized trials to compare a C3Link program with enhanced usual care. This study aims to (1) conduct a cluster randomized trial to assess short-term (intervention phase) and longer-term impact (maintenance phase) of the C3Link package of strategies, (2) use a mixed methods approach to assess multilevel implementation outcomes, and (3) perform cost-effectiveness and return on investment analysis to support scale-up of effective approaches and strategies.

### Hypothesis Testing

We will test the following hypotheses: (1) women from communities with more evidence-based strategies (C3Link Plus followed by C3Link Core and then enhanced standard of care) will have progressively higher cancer screening uptake (completion of recommended breast and cervical screenings); (2) women from communities with C3Link Core or Plus strategies will have higher follow-up compliance with supplemental or diagnostic follow-up referrals and initiation of cancer treatment than women in the enhanced standard of care; (3) women from C3Link Plus communities will have higher completion of recommended repeat breast and cervical cancer screenings than women from C3Link Core or enhanced standard-of-care communities; and (4) women from communities that receive C3Link Core or Plus strategies will have improved self-efficacy, social support, and knowledge of cancer risk factors compared to women in the enhanced standard-of-care communities.

## Methods

### Research Design

We will adopt a pragmatic study design to systematically assess implementation strategies to increase breast and cervical cancer screenings and evaluate the sustainability of the strategies. Due to multiple multilevel barriers associated with the extremely low screening uptake in Kenya, we created the C3Link multicomponent strategies targeted at barriers at the individual, interpersonal, and structural levels. These strategies are guided by the COM-B change model [3] as described in the Introduction section.

### Selection of Study Counties and Facilities

The C3Link trial will be conducted in 3 counties in Kenya: Machakos, Nakuru, and Nyeri. We will target populations inhabiting fixed dwellings in neighborhoods that are within breast and cervical cancer screening clinic catchment areas. Communities will be within a 30-minute walking radius from the clinics and a 90-minute travel radius from mammography facilities.

In consultation with key stakeholders in cancer control, we purposively selected the 3 representative counties. In each county, we identified 3 primary care clinics that offer breast and cervical cancer screenings. Each of the study clinics have more than 3 similar neighborhoods (community health units) attached to it. Our goal was to select distinct communities that were not contiguous to avoid cross-contamination.

### Implementation Strategies

Women enrolled in the C3Link Core and Plus communities will receive a package of strategies delivered by study CHWs in the community setting. CHWs are community members who receive standardized training to collect data on demographics and health needs in their communities while supporting management of minor ailments and making referrals for other ailments. Each CHW supports up to 100 households. In Kenya, they are referred to as community health promoters.

CHWs play an important role in addressing individual and interpersonal barriers to improve knowledge and encourage women to seek medical care. In order to optimize available resources, we will adopt a tiered process through which only women who require multiple strategies are offered them. We will include a C3Link Core group receiving education sessions only where CHWs deliver group education sessions to women and their support persons, a C3Link Plus group will receive the C3Link Core group education sessions and later add on individual evidence-based strategies that increase in resource intensity in a sequential order, targeting women who remain unscreened at 1-month intervals, and a third group receiving enhanced usual care (ie, distribution of flyers in addition to usual care).

At the clinic level, C3Link Core and Plus strategies will use practice facilitation in clinics to foster team-based care with CHWs embedded within the clinic teams to build optimal community linkages and communication channels. CHWs serving as team members in the clinic setting can cocreate optimal processes in screening clinics to coordinate the return of test results to women and support completion of follow-up recommendations. We will provide education to health care providers to improve communication and reduce any stigmatizing behaviors. Through the practice facilitation process, we will implement a learning collaborative that offers peer-to-peer consultations to improve screening capacity.

The enhanced standard-of-care communities will not have any CHWs. During enrollment, we will distribute campaign flyers provided by the National Cancer Institute of Kenya. Women who visit clinics may benefit from quality improvement processes. Table 1 provides details on the activities and goals of strategies during each phase of this study.

**Table 1.** Training and delivery of the multicomponent strategies.

Setting—Goal	C3Link^a^ Core and C3Link Plus	Enhanced standard of care
Preimplementation phase for planning (6 months)		
Community—train CHWs^b^	13 basic modules plus 6 modules of study-specific content	No study CHWs
Clinic—strengthen quality, referrals, and provider interactions	Readiness assessment; standard procedures, diagnosis triage; training on high-quality screening and documentation	Readiness assessment; standard procedures, diagnosis triage; training on high-quality screening and documentation
Intervention phase to test multicomponent strategies (24 months implementation and follow-up)		
Community— Increase women’s knowledge, self-efficacy, care-seeking behavior, and social support (reduce stigma) Deliver individual strategies that increase in intensity to women who need additional support (C3Link Plus only)	C3Link CHW-led education sessions (each community): Three-weekly women’s club meetings with education modules Six group sessions for family members or support persons	Awareness campaign in the community using flyers developed and provided by the National Cancer Institute of Kenya
Community— Increase women’s knowledge, self-efficacy, care-seeking behavior, and social support (reduce stigma) Deliver individual strategies that increase in intensity to women who need additional support (C3Link Plus only)	C3Link Plus only—focus on care-seeking behavior: Level 1: women not screened at 9 months will receive one-on-one education, level 2: women not screened at 10 months will receive individual counseling through motivational interviewing, and level 3: women not screened at 11 months will receive navigation to address barriers	Awareness campaign in the community using flyers developed and provided by the National Cancer Institute of Kenya
Clinic— Embed study CHWs so that, with their support, results and referrals are conveyed Improve the quality of screening	Practice facilitation with community linkages (each clinic) Monthly case review meetings with study CHWs and PDSA^c^ cycles with clinic teams led by clinic champions	Those who visit clinics may benefit from improvements, but there are no community–clinic linkages with CHWs
Maintenance phase to test reduced level of support (additional 24 months for a total of 36 months of follow-up)		
Community—provide support to maintain behavior change	Quarterly women’s club meetings Two group sessions for family members or support persons	Awareness campaign continues
Clinic—further embed team-based care and linkages	Quarterly meetings with study CHWs and PDSA cycles	Individuals may benefit from quality improvements at visits

a CHW: community health worker.

b C3Link: cancer community–clinic linkage.

c PDSA: Plan-Do-Study-Act is a rapid-cycle improvement process.

### C3Link Community Implementation

We will recruit C3Link study CHWs (women) from the C3Link communities in the preimplementation phase using the same process used by the Kenyan counties. We will assign 1 study CHW in each community selected to implement C3Link Core or Plus strategies. To ensure continuity, the study CHW will have the primary responsibility for the implementation and will be supported by county CHWs who will step in if required. The study CHWs will receive a 1-week training session that will be delivered by our study team. They will receive training on 13 standardized topics, including practical experience providing services in the community. The study-specific topics will include breast and cervical cancer screening, review of the content of the education sessions for women and support persons, and facilitation of group education sessions. The C3Link Plus CHWs will later receive training on the facilitation of one-on-one education sessions, motivational interviewing techniques, and delivery of navigation services. The C3Link study-specific training content will be prepared by updating training materials (based on published toolkits) [49-53] developed by the team for ongoing studies (R01CA200845 and UH3HD096908). Study CHWs will also be trained in the use of the C3Link CHW tracking tool, in which they will be documenting all interactions with the participants.

### CHW–Led Group Education Sessions for Women and Family Members

The education modules for women will be based on training materials and graphics developed by the Kenyan MOH for the pilot studies conducted in Nyeri and Meru counties [14,31]. Modules related to stigma and decision-making will be drawn from toolkits previously used in the Kenyan setting and available in both English and Kiswahili [54,55]. We will convene focus groups of 10 participants in each county (outside the study communities) to pretest the education modules for comprehension, appropriateness, and understandability.

The education modules will include 12 modules for women packaged into 2-hour women’s club or support person sessions. For women, the core content will be delivered during the first 9 months of the intervention phase, and additional content will be offered for the remaining 12 months. The 6 group education sessions planned for caregivers will cover the core content and will take place every other 3 weeks during the first 9 months of the intervention phase. During the maintenance phase, the reduced number of education sessions for women and caregivers will be drawn from the additional content that is available and tailored to the needs articulated by participants.

### C3Link Plus Sequence of Personalized Strategies

A sequence of individual strategies will be provided by study CHWs to women who remain unscreened at the 9-month follow-up. Women will be considered unscreened if they do not initiate any screenings or complete only one of the recommended screenings (breast or cervical). First, at 9 months, one-on-one education to reinforce concepts from group sessions and address specific misconceptions or myths expressed by the women. Second, those who remain unscreened at 10 months will receive motivational interviewing. Third, those who remain unscreened at 11 months will receive navigation to address any remaining barriers.

### Women Requiring Follow-Up Procedures

Study CHWs will inform participants within communities when HPV test results become available. During the HPV test results collection and breast cancer screening visit, providers will inform participants who require diagnostic procedures or treatment. Otherwise, for women who will not go to the clinics for their results and/or breast cancer screening, study CHWs will contact them after 2 weeks—encouraging them to visit their respective clinics, and providing navigation to support completion of required tests or treatments as needed. As in prior pilot studies, these procedures will be financed through Kenya’s Social Health Authority with supplementation from this study when necessary.

### C3Link Clinic Setting

During the preimplementation phase, we will conduct a readiness assessment to deliver breast and cervical cancer screening at the 9 participating clinics using the World Health Organization’s Service Availability and Readiness Assessment [56,57], which has been previously used to evaluate cancer screening capacity. This will help the team assess screening procedures, staffing, and supplies to identify potential gaps that would be addressed before implementing the multicomponent strategies. As part of this process, we will review standard operating procedures (SOPs) for the screening process, data entry in clinic tracking systems, and referrals for supplemental and diagnostic testing and cancer treatment. To evaluate the implementation of the SOPs and identify potential bottlenecks, we will conduct a pilot study by offering cancer screening and follow-up services to 10 eligible women from the catchment area of 1 participating clinic in each county. These 30 women will be selected from communities not included in this study, and lessons learned will be used to finalize the SOPs and improve clinic data quality. We will host a 1-day training in each county for physicians and nurses—2 health care providers per clinic, to strengthen workforce capacity on practice facilitation to deliver high-quality breast and cervical cancer screenings, improve patient communication, and reduce stigmatizing behavior [58]. The clinic team will receive training using existing training materials on the following topics [59-61]: (1) running effective meetings and supporting team discussion, (2) developing a formal process to seek updates from C3Link CHWs and clinic teams to improve patient communication, (3) reviewing screening and referral data with the team, and (4) using the Plan-Do-Study-Act method [61] to address any gaps in implementing the SOPs.

### Study Design and Sample Size Determination

**Figure 2.**
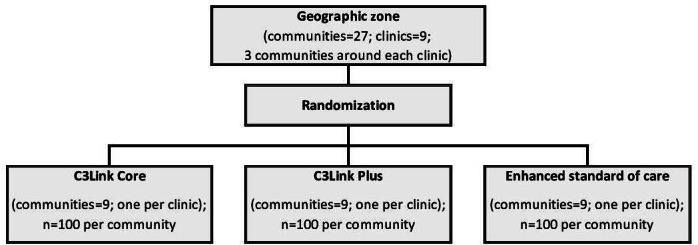
Cluster randomization for the cancer study. C3Link: cancer community–clinic linkage.

The sample size was determined using standard parameters (*α*=.05, 2-sided tests), based on the primary end point and allowing for the detection of a 15 percentage point improvement in the primary end point, our minimum threshold to consider the C3Link package of strategies to be successful. For the enhanced standard-of-care arm, given the current low level of screening, we estimated the screening uptake for both cancers to be about 15% at the 24-month follow-up (end of intervention phase). With a sample size of 100 in each of the 9 communities in the trial arms (for a total of 2700 women, and each woman will select 1 family member or support person), we will be able to detect a 15% difference with over 80% power, even after accounting for up to 20% attrition at 24 months. We selected a screening uptake of at least 30% in either the C3Link Core or Plus arm on the basis of prior study results [62,63]. We estimate that the interclass correlation coefficient would be in the range of 0.03 to 0.05 because interclass correlation coefficients for binary data are typically quite small, especially when screening uptake is very low [64]. Furthermore, we anticipate that the C3Link Plus arm would achieve a higher uptake than the C3Link Core arm, and we would be able to detect any difference of 15 percentage points between this study’s arms (ie, screening uptake of 15%, 30%, and 45% in the enhanced standard-of-care, C3Link Core, and C3Link Plus arms, respectively) with over 90% power. In each community, we will select study participants, 1 woman per household, using the Kish method [65], with eligibility remaining the same as previously stated. We will oversample by 10% to account for participants who cannot be contacted, refuse to participate, or are no longer eligible. The C3Link study cluster randomization is presented in Figure 2.

### Recruitment

The 3 communities per study clinic will be randomly assigned to one of the following study arms: C3Link Core, C3Link Plus, or enhanced standard of care. Trained study data collectors and study CHWs will visit households in the selected communities with the help of county CHWs (promoters). They will visit households in selected communities and enroll eligible women. Women will be eligible to participate if they are 30 to 55 years of age to capture most cases of persistent HPV infections (lower age limit) and those within the peak age groups of breast cancer incidence rate in Kenya (higher age limit). These women will be due to receive both breast and cervical cancer screening during this study’s intervention phase based on Kenyan guideline recommendations, not experiencing breast or cervical cancer symptoms, not pregnant, fluent in a study language (English, Kiswahili, Kamba, Kalenjin, and Kikuyu), and have plans to remain in the community for at least 2 years. We will include women who meet the inclusion criteria (1 per household using the Kish method) [65].

The study CHWs will administer a short survey to all women to assess eligibility for this study. The data collectors will administer the baseline survey to women who will give voluntary informed consent to participate. Each enrolled woman will be allowed to select a support person (spouse or community member) older than 18 years of age—the legal age for consent for health research in Kenya, with help from their CHW if needed. Selecting a support person will be voluntary. Study CHWs will administer a support person eligibility checklist tool before data collectors administer the support person baseline survey to those who give voluntary informed consent.

### Data Collection

To assess the impact of the C3Link Core and Plus strategies on the mediating outcomes, we will conduct surveys to collect data from women, support persons, and providers in the clinics. Study CHWs will document strategy implementation in the C3Link CHW tracking tool, and this information will be used to determine the “dose of the intervention” (ie, in the community setting, dose is the number of education sessions attended or the type of individual-level intervention). We will track all health care service use of women enrolled in this study by conducting clinic audits at all 9 clinics and referral facilities. Data will be collected using tablets, which will contain REDCap (Research Electronic Data Capture; Vanderbilt University) programs to collect and store the data. Deidentified data will be transferred and stored in secure, password-protected servers. Research Triangle Institute (RTI) and Implenomics staff will conduct further data quality review, produce reports, and develop analytic files for collaborative analysis. Tables2  3 present end point definitions and biological, imaging, self-report, and clinic audit data collected at baseline and to be collected at 12-, 24-, and 36-month follow-ups.

**Table 2.** Data collection at baseline, 12 months, 24 months, and 36 months. Data elements for primary, secondary, and follow-up end points—cancer care continuum.

Constructs or measures	Instrument and specification	Source	Timing
Proportion of women undergoing recommended cancer screening (primary end point measured at 24 months)	Cervical: Papanicolaou test (cytology laboratory), HPV^a^ DNA (Roche Cobas platform or Cepheid GeneXpert); breast: CBE^b^ or mammograms	Biological, imaging, and clinical procedures identified through clinic audits and this study’s database	12, 24, and 36 months
Proportion completing supplemental and diagnostic procedures in ≤90 days	Measured as days from referral to undergo procedures such as ultrasound, biopsy, colposcopy, and ablation of cervical lesions	Biological, imaging, and clinical procedures identified through clinic audits and this study’s database	24 and 36 months
Proportion of patients with cancer diagnosis initiating treatment in ≤90 days	Measured as days from cancer diagnosis to start of treatment; prespecified abstraction tool to identify dates and procedures	Biological, imaging, and clinical procedures identified through clinic audits and this study’s database	24 and 36 months
Proportion of patients completing repeat screening at the recommended interval (at 36 months)	Repeat screening based on Kenya guidelines and risk profile of the women, prespecified abstraction tool	Biological, imaging, and clinical procedures identified through clinic audits and this study’s database	36 months

a HPV: human papillomavirus.

b CBE: clinical breast examination.

**Table 3.** Data collection at baseline, 12 months, 24 months, and 36 months. Mediating outcomes, auxiliary end points, intervention dose, and clinic-based services.

Demographics and socioeconomic status	RTI^a^ cervical and breast cancer Kenya survey	Self-report	Baseline
Self-efficacy (women)	Generalized Self-Efficacy Scale [66]	Self-report	Baseline, 12 months, 24 months, and 36 months
Social support (women and family)	Brief Social Support Questionnaire [67], frequency of contact	Self-report	Baseline, 12 months, 24 months, and 36 months
Cancer stigma (women and family)	Perceived and anticipated [27,68]		Baseline, 12 months, 24 months, and 36 months
Cancer risk (women and family)	Breast Cancer Awareness Measure [69], RTI Kenya Survey	Self-report	Baseline, 12 months, 24 months, and 36 months
Self-care and care-seeking behavior, including wellness care (women)	Proportion conducting breast self-examination, number of visits, proportion with blood pressure and blood sugar testing	Self-report clinic audit	Baseline, 12 months, 24 months, and 36 months
Confidence in provider or system (women)	RTI Kenya survey questions on access and quality of care	Self-report	Baseline, 12 months, 24 months, and 36 months
Group education (women and family)	Number and proportion of education sessions attended (dose)	Study CHW^b^ tool study database	12, 24, and 36 months
Individual strategies (C3Link Plus only)	Number of women receiving each level of strategy (dose)	Study CHW tool study database	12, 24, and 36 months
Clinic team training sessions	Proportion of patients who attended the training sessions (dose)	Study CHW tool study database	12, 24, and 36 months
Clinic team meetings (study CHW and provider)	Number and proportion of meetings attended (dose)	Study CHW tool study database	12, 24, and 36 months
Clinic staff knowledge and attitudes (20 providers per clinic initially trained)	Tailored survey on screening quality, skills from training, and patient communication; stigma (modified Nyblade instrument) [70]	Self-report	Baseline and 24 months
Quality of screenings and communication of findings	Number of inadequate cervical samples (self and provider collected); proportion of mammography reports with breast density; percent test results shared with women within 2 weeks	Clinic audit, study CHW tool, and study database	Monthly
Screening for breast and cervical cancer	Number and type of tests; proportion of women receiving the test	Clinic audit, study CHW tool, and study database	12, 24, and 36 months
Additional procedures by screen type	Number of supplemental and diagnostic tests conducted	Clinic audit, study CHW tool, and study database	12, 24, and 36 months

a RTI: Research Triangle Institute.

b CHW: community health worker.

### Data Analysis

Hypothesis testing will be carried out by intent to treat, and we will examine the effects at the individual level across the 3 randomization groups. We will use generalized estimating equation models to estimate the effects of the randomization groups. If the Hausman assumption of correlation between the random and fixed effects is violated, then we will include fixed effects representing cluster identification [71]. We will adjust for baseline covariates, including sociodemographic and behavioral factors, should the initial descriptive analyses suggest differences in the distribution of these factors across study randomization groups. We will also explore the use of propensity scores to control for systematic differences between the groups. In further analyses, we will compare differences in mediating outcomes across the groups and examine the potential moderating roles of key individual behavioral, social, and structural factors hypothesized to influence cancer screening and adherence along the cancer care continuum, as shown in Figure 1. We will test the “dose” of the C3Link Core and Plus strategies received as a covariate in these models. We will explore differences in breast and cervical cancer screening completion and report the proportion that complete at least one of the screenings. As appropriate, we will perform analyses separately or pooled together for the younger and older cohorts of women to assess whether care-seeking behavior differs by age group. A key aspect of interest in this study is the extent to which perceived and anticipated stigma affects care-seeking behavior and screening completion. In subsequent analyses, we will consider both medium-term outcomes within the first 12 months after enrollment and changes at any point during the 24-month and 36-month follow-up period to assess longer-term outcomes. To understand potential variation across communities, we will conduct comparisons to analyze differences in mediating and screening outcomes among each of the C3Link Core and Plus communities. We will also report on the overall quality of the screenings provided, women’s preferences for cervical cancer screening, and the type of procedures used along the continuum of care. For the C3Link Plus arm, we will document the increase in screening completion with each step in the series of individual strategies and the number of women requiring each type of strategy.

### Assessing Multilevel Implementation Outcomes Using a Mixed Methods Approach

The objective of this assessment is to seek feedback from those implementing and receiving the strategies to evaluate the process of implementation. Data collection for this assessment will take place at 12 months post start of the intervention. We hypothesize that the C3Link Plus package of strategies will be more complex to implement in the community setting than the C3Link Core; therefore, we will compare the implementation outcomes between these 2 study arms to inform the selection of combination strategies. We will collect feedback from stakeholders across the 18 C3Link Core and Plus communities using a set of qualitative measures. The explanatory sequential design will strengthen the validity of quantitative data through triangulation and support the development of rich conclusions and actionable policies.

### Quantitative Measures

We will collect measures as outlined in Table 4 for both C3Link Core and C3Link Plus strategies.

**Table 4.** Implementation outcomes measurement.

Constructs	Measures	Stakeholder and data	Frequency
Acceptability, feasibility, and appropriateness	AIM^a^, FIM^b^, and IAM^c^ 4-item measures	Women, family, study CHW, and clinic team surveys	24- and 36-month follow-up
Fidelity^d^ (group education, clinic team meetings, and individual strategies)	All content or topics are delivered, and sessions and meetings take place in the frequency and sequence planned	Study CHW tracking tool and clinic meeting minutes	12, 24, and 36 months
Sustainability	SMSS^e^ subscales specific to strategies	Study CHW and clinic team survey	24 and 36 months

a AIM: Acceptability of Intervention Measure.

b FIM: Feasibility of Intervention Measure.

c IAM: Intervention Appropriateness Measure.

d Dose is also included, and details are provided in Tables2  3.

e SMSS: Sustainment Measurement System Scale.

Our measurement will include the 4-item questions from each of the Acceptability of Intervention Measure, Feasibility of Intervention Measure, and Intervention Appropriateness Measure [72]. We will collect implementation feedback from C3Link CHWs and clinic teams participating in team meetings. The fidelity measures described in Table 4 will be compiled annually throughout this study’s implementation, and summary statistics will be generated to assess compliance with study protocols. Sustainability will be assessed using subscales of the Sustainment Measurement System Scale [73] applicable to the CLink package of strategies.

### Qualitative Feedback

We plan to conduct interviews and focus groups to identify underlying moderating factors associated with the successful implementation of strategies, along with facilitators and barriers to inform future scale-up activities. First, at the end of the intervention implementation phase (24 months), trained data collectors will interview all 18 study CHWs from the C3Link Core and Plus communities and 6 clinic team members participating in C3Link implementation activities in each of the 9 clinics. These 18 CHWs are the total number of C3Link CHWs, where 9 are from the C3Link Core arm, and 9 are from the C3Link Plus arm. These CHWs will be distributed equally among the 3 counties—6 per county. We anticipate that differences and similarities will be based on study arm, county, or on the different iterations of the 2 variables. Categorization by study arm, by county, and by the different iterations may enrich the stories, but data saturation may be compromised. This may be reduced by the fact that participants will be information-rich, having been involved in the implementation continuously for over a year. We will develop an interview guide with questions and probes to seek feedback from study CHWs and clinical teams on their level of preparedness to implement the strategies, their assessment of the quality of the delivery, and the level of responsiveness of the target groups. We will also pose open-ended questions to determine what they felt were facilitators to deliver the strategies and potential barriers. In follow-up questions, we will explore suggestions to address the barriers.

Second, at the end of the maintenance phase (36 months), the research team will conduct 6 focus groups with C3Link CHWs and clinic teams that were interviewed (2 focus groups per county). These focus groups will explore topics related to the sustainability of the package of strategies, drawing on the findings from the Sustainment Measurement System Scale survey results. The focus group setting will allow for discussion among these key stakeholders and offer concrete feedback for future implementation in terms of best practices to further optimize the delivery of the strategies. To facilitate analysis, all interviews and focus groups will be transcribed and translated with supervision from the Kenya grant implementation team and stored in secure servers.

### Qualitative Data Analysis

Using coding reliability thematic analysis [74,75] and keeping an audit trail of coding decisions and theme development, 2 grant analysts will independently review the transcripts to develop the initial codebook. Transcripts will be coded and key themes derived using qualitative software such as NVivo (Lumivero). An iterative process of rereading the transcripts may identify new codes, and the codebook will be modified accordingly. All investigators will critically discuss interpretations of the data, with focus on accuracy and consistency, until they reach a consensus on the dominant themes and meanings, minimizing analysts’ subjectivity and bias that is based on their own background and understanding, as would be seen in reflexive thematic analysis [74]. Important excerpts will be included in reports and publications. Qualitative findings will offer insights into differences in the delivery of strategies in the C3Link Core and Plus arms.

### Triangulation of Findings

We will compare the quantitative data with the qualitative findings to identify areas of convergence and divergence. The study team will review the tabulated data on the implementation outcomes with the key themes drawn from the interviews and focus groups with study CHWs and the clinic team members. Areas of agreement will be highlighted, and we will use the qualitative data to provide additional nuanced context for the quantitative findings. When we identify discrepancies between the quantitative data and qualitative feedback, the study team will use reflective thinking based on learnings gained during the course of implementing the interventions to understand reasons for the variation. We will also discuss these findings with study counties to gain additional insights and to derive actionable findings for future implementation.

### Cost-Effectiveness and Return on Investment Analysis to Support Scale-Up of Effective Approaches and Implementation Strategies

The objectives of this analysis are the following: (1) to assess the short-term cost-effectiveness of C3Link Core and Plus strategies by conducting a comparative cost-effectiveness analysis across the trial arms and (2) to project the long-term cost-effectiveness of integrated approaches to promote breast and cervical cancer screenings. We will use a customized 3-step process to address these objectives.

### Step 1: Short-Term Cost-Effectiveness Analysis

As presented in Table 5, we will collect detailed cost data, and for the planned short-term analysis, we will generate the cost of start-up activities to understand the resources required to plan future implementation and the activity-based cost of strategies during the intervention and maintenance phases. We will use a previously validated instrument, the Cost Assessment Tool [76], to collect resource use and cost information. Our main goal is to estimate the implementation cost from the program perspective. We will estimate labor hours and document the expenditures on nonlabor resources to host trainings and women’s club and family education sessions, the cost of addressing barriers identified in C3Link Plus navigation services, and costs borne by women to undertake screenings. Based on these cost estimates and the screening completion end points, we will conduct an incremental cost-effectiveness analysis by generating the cost per woman screened. Using standard economics methodology [77,78], we will explore economies of scale related to implementation costs that can be achieved during scale-up.

**Table 5.** Cost data collection and analysis by phase.

Phase	Data category	Purpose	Data elements (examples)
Preimplementation phase	Start-up costs	To assess the cost of planning to implement strategies	Formalizing plans for the intervention; hiring staff; conducting training, needs assessment, and capacity building
Intervention and maintenance phases	Implementation of activity-based cost data	To assess the cost of implementing and maintaining interventions	Implementation activities: delivering strategies; tracking outcomes, quality assurance, data collection, and evaluation Patient cost: travel cost, time lost from work
Intervention and maintenance phases	Resource use	To standardize cost	Labor hours (study CHWs^a^ and clinic teams), nonlabor resources
Future expansion phase	Scale-up cost	To estimate the cost of large-scale implementation	Fixed vs variable activity-based cost estimates of implementation strategies to project economies of scale

a CHW: community health worker.

### Step 2: Cohort-Based Microsimulation Modeling for Breast and Cervical Cancer Separately

The screening effectiveness data and information on the use of services along the cancer care continuum from each of the trial arms will be used as inputs in previously validated breast and cervical cancer screening lifetime simulation models developed by RTI [79-81].

These models will be recalibrated with up-to-date input data from Kenya, including mortality and treatment survival statistics, and external validation will be conducted by comparing the outputs of the models (not including any interventions) with the current Kenyan stage at diagnosis and cancer incidence. We will conduct extensive sensitivity and uncertainty analysis to offer insights into likely real-world impacts [82]. The model results per 100,000 women will be used to compare differences across the enhanced standard of care, C3Link Core, and C3Link Plus. We will also generate cancer and mortality incidence rates under various screening scenarios in 5-year age increments for each trial arm.

### Step 3: Population-Level Simulation of the Impacts of Integrated Breast and Cervical Cancer Screenings

We will use data from the Kenyan census and the outputs from the microsimulation models to conduct population-level simulations of the joint impacts, considering both effectiveness and cost, of integrated promotion of breast and cervical cancer screenings. The cost information related to the integrated implementation of strategies for breast and cervical cancer screening from step 1, along with the cost of the screening tests, supplemental and diagnostic procedures, and cancer treatment from a recent publication by the research team [82], will be used to generate cost-effectiveness estimates. Our estimates will include both direct and indirect costs. In addition to the comparison across scenarios, we will compare the integrated promotion of breast and cervical cancer screening to the comparator of stand-alone screening programs for which we will derive projected cost and effectiveness data from published studies [62,83-85], to conduct scenario analysis. We will evaluate the trade-off, or return on investment, between the strategies used in each arm of the trial and report the projected decrease in cancer incidence and mortality under each set of strategies. We will conduct policy simulations to assess the impact of scaling up the interventions, perform sensitivity analysis, and generate potential best- and worst-case projections. This cost-effectiveness analysis will be complemented by a budget analysis, which will identify the financial outlays for the integrated strategies that will be required annually to implement the C3Link Core and C3Link Plus during various phases in the scale-up process.

### Ethical Considerations

This study is registered on ClinicalTrials.gov (NCT06572774), and deidentified data from this study (with consent from participants) will be updated within the registered trial. This protocol was reviewed and approved by the RTI (TUDY00022177) and Strathmore University Institutional Scientific and Ethical Review Committee (SU-ISERC1754/23), independent institutional review boards (IRBs), with plans to communicate any protocol amendments to them for approval.

Study staff were responsible for obtaining written informed consent from all participants. Before implementation, all informed consent forms in all study languages (English, Kiswahili, Kamba, Kikuyu, and Kalenjin) were approved by this study’s IRBs. In Kenya, the legal age for consent for health research is 18 years. A travel and snack compensation of 500.00 Kenya shillings (US $3.89) will be offered to women participating in the RCT, and 1000.00 Kenya shillings (US $7.78) will be offered to women participating in the pretesting of education materials and pilot testing screening procedures.

While many of the data collectors have previous research experience, they will undergo a 3-day training program on study procedures, data collection, and research ethics before the start of participant interaction, consent, and data collection activities.

In addition to study staff, we will establish a Data Safety Monitoring Board–independent from the sponsor and funder for quarterly monitoring meetings, which includes 3 Kenyan experts (an academic, a clinician, and a civil society representative) working on women’s cancer screening and care.

## Results

This study was funded in July 2022. IRB approvals were in November 2022 (RTI IRB) and in June 2023 (Strathmore University–Institutional Scientific and Ethical Review Committee IRB). This study was registered on ClinicalTrials.gov in August 2024. Recruitment of this study’s cohort began in September 2024 and was concluded in December 2024. For timeline context, the cohort will be followed for 36 months, and data will be collected annually. Our primary end point analysis related to screening uptake and follow-up diagnostic test completion will occur after the collection of our 24-month data collection (in early 2027). We are conducting ongoing fidelity assessments of our interventions and reviewing the quality of data as we progress with this study’s implementation. We will host multiple forums with policymakers at the MOH, The National Cancer Institute of Kenya, and the counties. The head of NCI Kenya will serve as a key liaison to disseminate findings. The results of all analyses will be reported in separate manuscripts.

## Discussion

### Defining Features of our Approach

We will assess the effectiveness and cost-effectiveness of multicomponent behavioral strategies to increase the uptake of breast and cervical cancer screening and completion of recommended diagnostic and treatment services of the C3Link program conducted in 3 counties in Kenya. Our strategies, including CHW-led group education sessions for behavior change [86,87] and practice facilitation at clinics [88], have been shown to increase screening uptake and recommended follow-up procedures among populations. The varied intensity of our implementation strategies with sequential increments allows for an efficient process to identify a combination of implementation strategies with available resource-based guidance, which addresses a key constraint in implementing strategies in low- and middle-income countries.

This is a novel approach to conduct a systematic economic evaluation of integrated strategies and delivery of screening to support scale-up. Currently, there is limited evidence on the cost, cost drivers, and cost-effectiveness of integrated promotion of strategies to increase screenings. Integration can potentially be more cost-efficient than siloed approaches, but policymakers need evidence to make informed decisions. We will use a 3-step process that will begin with the assessment of the short-term cost-effectiveness of the 2 C3Link packages of strategies investigated in the cluster randomized trial, followed by cohort-based microsimulation modeling of the long-term impacts on incidence and mortality generated separately for breast and cervical cancers using existing models designed by the grant team [79-81]. Finally, we generate population-level projections of the budget impact and mortality reduction of integrated screenings.

We will create and maximize unique partnerships for sustainability, with stakeholder engagement and evidence from previous studies to co-design, plan, implement, and evaluate the C3Link strategies. We will work with county teams at each step of this study to ensure continual collaboration and community participatory approaches. Our goal is to develop county-level research and implementation capacity to strengthen cancer screening delivery beyond the grant period. Partnerships with governmental and community stakeholders promote adaptation, uptake, scalability, and sustainability of evidence-based interventions [89].

Although cluster randomization reduces contamination across study arms, it increases the risk that women in each arm may differ at baseline. This study’s sampling frame will allow us to assess baseline differences before randomization and adjust our sampling process with propensity score weighting methods. Second, data could be missing because of nonresponse and study attrition. This study’s CHWs in each community will maintain regular contact with the women, and we will also use rigorous field data collection practices, including training data collectors, developing protocols, and monitoring fidelity continually. To limit missing data, we will train clinic staff and data collectors and implement continuous quality review. Furthermore, we will address any missing data by including demographic covariates that will serve as proxies for dropout and conducting sensitivity analyses.

We will select 1 eligible woman from within each household in the selected communities. To reduce interviewer bias, we will use the Kish method to provide a random and consistent procedure for women’s selection.

A key constraint in implementing strategies in low- and middle-income countries is funding. This is the first cancer screening study in Kenya, and to our knowledge, in any other limited-resource setting, that will evaluate implementation strategies in a predetermined sequence and assess effectiveness and cost at each step. This will allow us to determine the incremental cost-effectiveness of each strategy in the sequence to guide policy. For example, given the available resources and cost-effectiveness profile, a Kenyan county may decide to implement C3Link Core with only the first level in the sequence of C3Link Plus. Importantly, the findings will guide future studies in testing additional combinations and sequences.

This trial features novel evidence-based strategies to increase uptake of breast and cervical cancer screening in variously resourced settings in a cost-effective process. We anticipate that the findings will be valuable not only in Kenya but also in other sub-Saharan African countries.

### Conclusions

The C3Link Program is expected to yield better screening and follow-up outcomes than the control (the enhanced usual care), but will require more resources.

## Supplementary material

10.2196/87850 Checklist 1 SPIRIT Checklist

10.2196/87850 Checklist 2 SRQR Checklist

10.2196/87850 Peer Review Report 1 Peer review report by the HPC - Health Promotion in Communities Study Section Review Group, National Cancer Institute (National Institutes of Health, USA).

## Abbreviations

C3Link: cancer community–clinic linkage
C3Linkages: cancer community–clinic linkages
CBE: clinical breast examination
CHW: community health worker
COM-B: Capability, Opportunity, and Motivation–Behavior
HPV: human papillomavirus
IRB: institutional review board
MOH: Ministry of Health
REDCap: Research Electronic Data Capture
RTI: Research Triangle Institute
SOP: standard operating procedure
