# Supplementary material for: Integrated Community-Clinic Model of Optimized Implementation Strategies to Increase Early Detection of Breast and Cervical Cancers in Kenya: Protocol for a Pragmatic Randomized Controlled Trial
Source: JMIR Res Protoc. 2026 May 18;15:e87850. doi: 10.2196/87850 (PMC13182773; doi:10.2196/87850)
Supplement: Peer Review Report 1 [file resprot-v15-e87850-s003.pdf]

SUMMARY STATEMENT
(Privileged Communication)

Application Number: **1 R01 CA269472-01**

Principal Investigator

SUBRAMANIAN, SUJHA

Review Group:	**HPC** **Health Promotion in Communities Study Section**
`	

Meeting Date:	**10/18/2021**	*RFA/PA:*	**PAR19-274**
Council:	**JAN 2022**	*PCC:*	**AJGH**
Requested Start:	**04/01/2022**		

Project Title:	**An integrated community-clinic model of optimized implementation strategies to increase early detection of breast and cervical cancers in Kenya**
SRG Action:	**Impact Score:28 Percentile:12**
Next Steps:	**Visit https://grants.nih.gov/grants/next_steps.htm**
Human Subjects:	**30-Human subjects involved - Certified, no SRG concerns**
Animal Subjects:	**10-No live vertebrate animals involved for competing appl.**
Gender:	**1A-Both genders, scientifically acceptable**
Minority:	**5A-Only foreign subjects, scientifically acceptable**
Age:	**3A-No children included, scientifically acceptable**

1R01CA269472-01 SUBRAMANIAN, SUJHA

RESUME AND SUMMARY OF DISCUSSION: The proposed research will assess the effectiveness and cost-effectiveness of strategies to increase the uptake of cancer screening and completion of recommended diagnostic and treatment services among women in Kenya. The rigor of prior research is strong and the panel found this application highly significant in its ability to inform multilevel approaches to increase cancer screening in Kenya. If successful, the project could be scaled to other low- and middle-income countries in Sub Saharan Africa. The investigative team has the necessary expertise and strong history of collaboration to successfully carry out this project. Reviewers highlighted the team's strong engagement of both Kenyan and U.S. partners as a major strength. The research environment is excellent with the necessary resources and in-country presence in Kenya to support completion of study aims. The panel found several strengths with the approach. The approach is informed by strong preliminary data and includes well-justified methods for each aim. The multilevel approach allows investigators to address barriers to screening at multiple levels. The panel did note some weaknesses with the approach. It was unclear to reviewers why two interventions conditions are proposed rather than one. How missing data will be addressed is not detailed. The project lacks information on how participants who test positive would be linked to care. The panel also felt the application lacked details on how it would build local capacity to ensure sustainability. These weaknesses were considered remediable and did not detract from the panel's enthusiasm for the application. Overall, findings are expected to have a high impact on multicomponent strategies to increase uptake of breast and cervical cancer screening in Kenya and other Sub Saharan African settings through community-clinic linkages.

DESCRIPTION (provided by applicant): Kenya has a high incidence of breast and cervical cancers, and most women are diagnosed with late- stage disease. Implementing effective programs to screen and detect these cancers at an early stage could substantially decrease the high mortality. Kenya has been increasing access to cancer screening, but less than one-sixth of eligible women have ever received breast and cervical cancer screening. The goal of the study is to assess the effectiveness and cost-effectiveness of multicomponent strategies to increase the uptake of breast and cervical cancer screening. A key gap that perpetuates the low screening rates in Kenya is the lack of community–clinic linkages. There is evidence that women are often unaware of the screening services offered in facilities, and, even among those who are aware, there is lack of motivation, anticipated or perceived stigma, and reduced self-efficacy to undergo screening. We will conduct a pragmatic cluster randomized trial to evaluate the screening outcomes, including screening uptake, diagnostic test completion, and treatment initiation and implementation outcomes to support scale- up. We will test two multicomponent packages of strategies: (1) Cancer Community–Clinic Linkage (C3Link) Core: Community health volunteer (CHV)-delivered group education for women and family members to increase screening uptake in the community setting and practice facilitation to improve the screening process and develop team-based care with CHV participation in the clinic setting; (2) C3Link Plus: C3Link Core strategies along with a sequential series of individual strategies that increase in intensity at 3-month intervals; women who remain unscreened will first receive one-on-one education; second, motivational interviewing; and, finally, navigation to address specific barriers. We will implement the study across 27 communities in Kenya to address the following three specific aims: Aim 1: Conduct a cluster randomized trial to assess short-term (intervention implementation phase) and longer-term impacts (maintenance phase) of the two packages of multicomponent strategies on breast and cervical cancer screening outcomes along the continuum of care compared to enhanced standard of care (communities receiving a messaging campaign only); Aim 2: Use a mixed-methods approach to assess and compare multilevel implementation outcomes of the package of strategies focusing on acceptability, feasibility, appropriateness, fidelity, and sustainability; Aim 3: Perform cost-effectiveness and return-on-investment analysis to support scale-up of an effective package of implementation strategies across

Kenya and other sub-Saharan African countries. The Kenyan Ministry of Health can use the findings from this study to design optimal approaches and plan investments in screening infrastructure to scale up implementation of guideline recommendations. The lessons learned can be applied to other sub-Saharan African settings.

PUBLIC HEALTH RELEVANCE: Kenya has a high incidence of breast and cervical cancers, and most women are diagnosed with late-stage disease. We will conduct a study to assess the effectiveness and cost-effectiveness of strategies to increase the uptake of cancer screening and completion of recommended diagnostic and treatment services. The findings can be used to design optimal approaches and plan investments in infrastructure to scale up implementation of breast and cervical cancer screening.

CRITIQUE 1

Significance: 3
Investigator(s): 1
Innovation: 2
Approach: 1
Environment: 1

Overall Impact: This study addresses a highly significant issue of low-uptake of breast and cervical cancer screening in Kenyan women and thus reduce incidence of late stage cancers evident in the population, which is highly prevalent despite the availability of a screening infrastructure. The goals of this study are to test effectiveness and cost-effectiveness of multicomponent strategies to increase the uptake of breast and cervical cancer screening and completion of recommended diagnostic and treatment services across 27 communities in Kenya. They will conduct a pragmatic cluster randomized trial to evaluate the screening outcomes, including screening uptake, diagnostic test completion, and treatment initiation and implementation outcomes to support scale-up. Aim 1 will test two packages of multi-component strategies compared to enhanced standard of care: a) community health volunteer delivered education, practice facilitation to improve CHV participation in the clinical setting, b) the previous strategies and individual level one-on-strategies that increase in intensity over time such as motivational interviewing and navigation for specific barriers. Aim 2 will use mixed methods to assess and compare multilevel implementation outcomes, and Aim 3 will assess cost-effectiveness at each level of implementation. The proposal has several strengths including multilevel approach and to address multi-level barriers and generating policy actionable data at multilevel. Team brings complementary expertise and strong record preliminary work in the study setting to support the feasibility of the proposal. The approach is community engaged, and the methods are appropriate and justified to meet the aims. Minor weaknesses include missing rigor of prior research outside of the work of the study team to support the premise of the application. The overall impact of the proposed study remains high due to the potential for considerable shift in cancer screening uptake with stakeholder engagement embedded at all levels of the multilevel approach and the examination of incremental cost-effectiveness at multiple levels to support policy uptake.

1. Significance:

Strengths

- The study is poised to address a highly significant issue of low-uptake of cancer screening in Kenyan women and thus reduce incidence of late stage cancers evident in the population.

- Prior research supporting this application includes preliminary results from multiple investigations by the study team in identifying multi-level barriers to cancer screening uptake, establishing community-clinic linkages to deliver integrated services, and demonstrating the feasibility of using community health volunteers to support screening uptake activities.
- The multi-level approach to intervention addresses multi-level barriers.

Weaknesses

- Rigor of prior research from the broader field to support justification of the premise of the approach absent from the proposal.

2. Investigator(s):

Strengths

- This is a strong investigative team with complementary expertise to carry out the proposed study.
- PI has expertise and a track record as an implementation scientist and economist, and has extensive experience with implementation studies in international settings.
- Team demonstrates multidisciplinary expertise with in-country and U.S. partners.

Weaknesses

- None noted by reviewer.

3. Innovation:

Strengths

- The study would be a considerable shift in cancer screening uptake with stakeholder engagement embedded at all levels of the multilevel approach and the examination of incremental cost-effectiveness at multiple levels to support policy uptake.
- Use of microsimulation modeling with the cluster-randomized trial to assess implementation outcomes provide an efficient means to generate evidence to impact policy and speed up implementation.

Weaknesses

- None noted by reviewer.

4. Approach:

Strengths

- The methods for each aim are appropriate and well justified.
- Establishment of a community advisory board to provide broad feedback on the study findings is a strength, including additional stakeholder engagement embedded throughout all aspects of the study.
- The implementation strategies of interest are evidence-based.
- The implementation strategies are comprehensive.
- Outcomes are appropriate.

- Procedures for deployments of implementation strategies and their assessment are well described.

Weaknesses

- None noted by reviewer.

5. Environment:

Strengths

- This is a well-resourced environment and infrastructure to carry out the proposed study.

Weaknesses

- None noted by reviewer.

Study Timeline:

Strengths

- Adequate.

Weaknesses

- None noted by reviewer.

Protections for Human Subjects:

Acceptable Risks and/or Adequate Protections

Data and Safety Monitoring Plan (Applicable for Clinical Trials Only):

Acceptable

Inclusion Plans:

- Sex/Gender: Distribution justified scientifically
- Race/Ethnicity: Distribution justified scientifically
- Inclusion/Exclusion Based on Age: Distribution justified scientifically
- none

Vertebrate Animals:

Not Applicable (No Vertebrate Animals)

Biohazards:

Not Applicable (No Biohazards)

Resource Sharing Plans:

Acceptable

Budget and Period of Support:

Recommend as Requested

CRITIQUE 2

Significance: 1
Investigator(s): 1
Innovation: 1
Approach: 4
Environment: 1

Overall Impact: Implementation science approaches to breast and cervical cancer screening in Kenya are critical and novel and may have implications for other countries in Sub-Saharan Africa. The PI from RTI and binational team and partners are well-prepared to conduct the proposed community-engaged project. The approach features a cluster RCT using mixed methods and multilevel interventions as well as assessment of sustainability and cost analyses to address scale up. There are some addressable concerns about the interventions and power for the selected outcomes. These are outweighed by the strengths of the project, which is likely to have a major impact.

1. Significance:

Strengths

- Breast and cervical cancer screening is minimal in Kenya, resulting in late stage diagnosis and high mortality.
- Kenya has made free screening available, but uptake is low.
- Multilevel barriers across the cancer continuum have been identified and will be addressed.
- What is learned here may be applicable to other Sub-Saharan countries.

Weaknesses

- None noted by reviewer.

2. Investigator(s):

Strengths

- PI and team have relevant experience and expertise.
- Binational team with local partnerships.
- Some of the team has worked together previously conducting relevant pilot work.

Weaknesses

- None noted by reviewer.

3. Innovation:

Strengths

- Rigor of the prior research: Little implementation science research on breast and cervical cancer screening has been conducted in Kenya/Sub-Saharan Africa.
- Cost analyses and microsimulation modeling to assess scale up and future impact.

Weaknesses

- None noted by reviewer.

4. Approach:

Strengths

- Multilevel, mixed methods, cluster randomized trial, cost analyses. Long term follow up, assessment of maintenance.
- Community-based. E.g., Women-centered wellness intervention addressing broader health issues as requested by the community.
- Existing validated implementation science measures have been selected for each study phase.
- Clinics and communities have been thoughtfully selected.
- Focus groups for the group intervention and a pilot study for the clinic setting logistics will be conducted prior to the cluster RCT.
- Figures and tables are helpful to understand models, structures, and methods.
- Study focuses on women's issues, but men will be assessed as appropriate.

Weaknesses

- Outcomes: The primary outcome of obtaining both screenings seems quite ambitious given the current low levels. The sample sizes for the secondary and follow up outcomes were not projected and may be quite small.
- Interventions: Unclear why three intervention conditions were chosen instead of two. The feasibility of women attending 12 groups and family members enrolling and attending 6 groups in the two C^3Link interventions was not addressed. There may also be contamination within the Plus intervention with CHVs mixing education, MI, or navigation.
- The potential for missing clinic chart data was not mentioned.
- The challenges of working in 5 languages was not discussed. Setting, personnel management, and logistics are complex and will be challenging. However, these are the foci of the project.

5. Environment:

Strengths

- RTI is well-prepared for this research, particularly with a local office in Kenya.
- Local partner is Kenya Medical Research Institute.
- Other partners include local investigators and government authorities.
- Relevant letters of support from consultants and local organizations were provided.

Weaknesses

- None noted by reviewer.

Study Timeline:

Strengths

- Acceptable

Weaknesses
- None noted by reviewer.

Protections for Human Subjects:
Acceptable Risks and/or Adequate Protections
- Acceptable

Data and Safety Monitoring Plan (Applicable for Clinical Trials Only):
Acceptable
- o External DSMB

Inclusion Plans:
- Sex/Gender: Distribution justified scientifically
- Race/Ethnicity: Distribution justified scientifically
- Inclusion/Exclusion Based on Age: Distribution justified scientifically
- Based on Kenyan breast and cervical cancer screening recommendations.

Vertebrate Animals:
Not Applicable (No Vertebrate Animals)

Biohazards:
Not Applicable (No Biohazards)

Applications from Foreign Organizations:
Not Applicable (No Foreign Organizations)
- Subcontract to Kenya setting

Resource Sharing Plans:
Acceptable

Budget and Period of Support:
Recommend as Requested

CRITIQUE 3

Significance: 2
Investigator(s): 1
Innovation: 3
Approach: 3
Environment: 1

Overall Impact: This is a strong application written by a senior research fellow at RTI International. The grant focuses on assessing the effectiveness and cost-effectiveness of multicomponent strategies to increase the uptake of breast and cervical cancer screening in Kenya. The proposed project is significant because: (i) it will help improve breast and cervical cancer screenings among Kenyan women; (ii) help reduce cancer related deaths in the country; and (iii) help reduce the multi-level barriers to cervical and breast cancer screenings. Currently, there is no such intervention in Kenya and if successful, findings from this project will help provide data on scaling up the intervention. The approach has a few strengths including (i) a well described components of the C^3Link and C^3 Link plus which have potential to increase cervical and breast cancer screenings; (ii) engagement of community health volunteers and utilization of inputs from CAB; (iii) use of community health volunteers as the people administering the intervention since they might be well known and trusted in their communities and, (iii) a well described cost effectiveness analysis plan. The investigative team is very strong and has the necessary skills to conduct the study. Strong letters of support from RTI International and the collaborating institutions. The main weakness of the approach includes (i) the process of linking those who test positive and helping them engage in care is not clearly explained - while screening for breast cancer and cervical cancer are very important, helping those who test to get treatment and remain in treatment is equally important and need to be clearly explained (ii) recruiting and retaining participants in the study might be difficult since there is no mention of compensation for the participants' time. Overall, the proposed project if successful will have a moderate impact in the field of breast and cervical cancers and be a model for other low- and middle-income countries with high incidence of these cancers but low screening rates.

1. Significance:

Strengths

- Breast and cervical screening is very low in Kenya with only 12% and 16% respectively
- Although women generally know about screening, they have limited understanding of the available cancer screening services therefore, this will help them access the screening.
- The proposed project can help reduce multi-level barriers to breast and cervical cancer screenings.

Weaknesses

- None noted by reviewer.

2. Investigator(s):

Strengths

- Dr. Subramanian has extensive experience in assessing the effect of integrated care delivery on early diagnosis and treatment of chronic conditions.
- The investigative team as a whole has the expertise to conduct the study.
- A multi-disciplinary team with wide background and experience related to the proposed project.

Weaknesses

- None noted by reviewer.

3. Innovation:

Strengths

- The proposed study is the first cancer screening study in Kenya, and in any other limited-resource setting, that will evaluate implementation strategies in a predetermined sequence and assess effectiveness and cost at each step.
- Opportunity to partner for sustainability with stakeholder engagement in a data-driven process to co-design, plan, implement, and evaluate the intervention.
- Novel approach to conduct systematic economic evaluation of integrated strategies and delivery of screening to support scale-up.

Weaknesses

- The opportunity for partnerships is well explained but capacity building for the local intuitions institutions is not clear.

4. Approach:

Strengths

- Strong evidence from preliminary study of the efficacy of the intervention.
- Engagement of the community health volunteers and involvement of interdisciplinary team at the clinic setting.
- Logical and measurable array of feasibility and acceptability.
- Cost effectiveness analysis to support scale-up.
- Formation and the use of input from the community advisory board ensure input from relevant stakeholders.

Weaknesses

- The process of linking those who test positive to treatment is not clear.
- It is not clear how participants will be compensated for their time to improve retention.

5. Environment:

Strengths

- Research Triangle Institute and the Kenya Medical Research Institute have appropriate resources to support the proposed project.
- Strong letters of support from Kenya local collaborators and intuitions.

Weaknesses

- None noted by reviewer.

Study Timeline:

Strengths

- The timeline is sufficient.

Weaknesses

- None noted by reviewer.

Protections for Human Subjects:

Acceptable Risks and/or Adequate Protections

- Risks are acceptable and protections are adequate

Data and Safety Monitoring Plan (Applicable for Clinical Trials Only):

Acceptable

- Adequate data safety and monitoring plan

Inclusion Plans:

- Sex/Gender: Distribution justified scientifically
- Race/Ethnicity: Distribution justified scientifically
- Inclusion/Exclusion Based on Age: Distribution justified scientifically
- Inclusion plan are appropriate

Vertebrate Animals:

Not Applicable (No Vertebrate Animals)

Biohazards:

Not Applicable (No Biohazards)

Budget and Period of Support:

Recommend as Requested

THE FOLLOWING SECTIONS WERE PREPARED BY THE SCIENTIFIC REVIEW OFFICER TO SUMMARIZE THE OUTCOME OF DISCUSSIONS OF THE REVIEW COMMITTEE, OR REVIEWERS' WRITTEN CRITIQUES, ON THE FOLLOWING ISSUES:

PROTECTION OF HUMAN SUBJECTS: ACCEPTABLE

INCLUSION OF WOMEN PLAN: ACCEPTABLE

INCLUSION OF MINORITIES PLAN: ACCEPTABLE

INCLUSION ACROSS THE LIFESPAN: ACCEPTABLE

COMMITTEE BUDGET RECOMMENDATIONS: The budget was recommended as requested.

Footnotes for 1 R01 CA269472-01; PI Name: SUBRAMANIAN, SUJHA

NIH has modified its policy regarding the receipt of resubmissions (amended applications).See Guide Notice NOT-OD-18-197 at https://grants.nih.gov/grants/guide/notice-files/NOT-OD-18-

197.html. The impact/priority score is calculated after discussion of an application by averaging the overall scores (1-9) given by all voting reviewers on the committee and multiplying by 10. The criterion scores are submitted prior to the meeting by the individual reviewers assigned to an application, and are not discussed specifically at the review meeting or calculated into the overall impact score. Some applications also receive a percentile ranking. For details on the review process, see http://grants.nih.gov/grants/peer_review_process.htm#scoring.

MEETING ROSTER

Health Promotion in Communities Study Section
Healthcare Delivery and Methodologies Integrated Review Group
CENTER FOR SCIENTIFIC REVIEW
HPC
10/18/2021 - 10/19/2021

Notice of NIH Policy to All Applicants: Meeting rosters are provided for information purposes only. Applicant investigators and institutional officials must not communicate directly with study section members about an application before or after the review. Failure to observe this policy will create a serious breach of integrity in the peer review process, and may lead to actions outlined in NOT-OD-14-073 at https://grants.nih.gov/grants/guide/notice-files/NOT-OD-14-073.html, NOT-OD-15-106 at https://grants.nih.gov/grants/guide/notice-files/NOT-OD-15-106.html, and NOT-OD-18-115 at https://grants.nih.gov/grants/guide/notice-files/NOT-OD-18-115.html, including removal of the application from immediate review.

CHAIRPERSON(S)

NICCOLAI, LINDA M, PHD
PROFESSOR
DEPARTMENT OF EPIDEMIOLOGY OF MICROBIAL DISEASES
YALE SCHOOL OF PUBLIC HEALTH
YALE UNIVERSITY
NEW HAVEN, CT 06520

MEMBERS

ARREDONDO, ELVA M, PHD
PROFESSOR
DEPARTMENT OF PSYCHOLOGY
SAN DIEGO STATE UNIVERSITY
SAN DIEGO, CA 92123

BERG, CARLA J, PHD
PROFESSOR
DEPARTMENT OF PREVENTION AND COMMUNITY HEALTH
GEORGE WASHINGTON UNIVERSITY
WASHINGTON, DC 20052

CONNER, KENNETH R, PSYD, MPH *
PROFESSOR
DEPARTMENTS OF EMERGENCY MEDICINE AND PSYCHIATRY
SCHOOL OF MEDICINE AND DENTISTRY
UNIVERSITY OF ROCHESTER MEDICAL CENTER
ROCHESTER, NY 14642

DANILOVICH, MARGARET K, DPT, PHD
RESEARCH SCIENTIST AND SENIOR DIRECTOR OF RESEARCH
LEONARD SCHANFIELD RESEARCH INSTITUTE
CJE SENIORLIFE
CHICAGO, IL 60645

DE HEER, HENDRIK DIRK, PHD, MPH *
ASSOCIATE PROFESSOR
DEPARTMENT OF HEALTH SCIENCES
NORTHERN ARIZONA UNIVERSITY
FLAGSTAFF, AZ 86011

EGAN, KATHLEEN LOUISE, PHD *
ASSISTANT PROFESSOR
DEPARTMENT OF HEALTH EDUCATION AND PROMOTION
EAST CAROLINA UNIVERSITY
GREENVILLE, NC 27858

GITTELSOHN, JOEL, PHD
PROFESSOR
DEPARTMENT OF INTERNATIONAL HEALTH
CENTER FOR HUMAN NUTRITION
BLOOMBERG SCHOOL OF PUBLIC HEALTH
JOHNS HOPKINS UNIVERSITY
BALTIMORE, MD 21205

HAGOBIAN, TODD ALAN, PHD *
PROFESSOR
DEPARTMENT OF KINESIOLOGY AND PUBLIC HEALTH
CALIFORNIA POLYTECHNIC STATE UNIVERSITY
SAN LUIS OBISPO, CA 93407

HECKMAN, CAROLYN J, PHD
ASSOCIATE PROFESSOR
ROBERT WOOD JOHNSON MEDICAL SCHOOL
RUTGERS CANCER INSTITUTE OF NEW JERSEY
RUTGERS UNIVERSITY
NEW BRUNSWICK, NJ 08901

HOWELL, KATHRYN H, PHD *
ASSOCIATE PROFESSOR
DEPARTMENT OF PSYCHOLOGY
UNIVERSITY OF MEMPHIS
MEMPHIS, TN 38152

ISLAM, NADIA S, PHD
ASSOCIATE PROFESSOR
DEPARTMENT OF POPULATION HEALTH
NEW YORK UNIVERSITY SCHOOL OF MEDICINE
NEW YORK, NY 10016

KEITA, AKILAH JOYCE, PHD
ASSOCIATE PROFESSOR
DEPARTMENT OF BEHAVIORAL AND SOCIAL SCIENCES
BROWN UNIVERSITY
PROVIDENCE, RI 02912

KOINIS MITCHELL, DAPHNE, PHD
PROFESSOR AND VICE CHAIR OF RESEARCH
DEPARTMENTS OF PEDIATRICS AND PSYCHIATRY
THE WARREN ALPERT MEDICAL SCHOOL
BROWN UNIVERSITY
PROVIDENCE, RI 02903

KRUKOWSKI, REBECCA A, PHD
ASSOCIATE PROFESSOR
DEPARTMENT OF PREVENTIVE MEDICINE
UNIVERSITY OF TENNESSEE HEALTH SCIENCE CENTER
MEMPHIS, TN 38163

LEE, SUNMIN, MPH, SCD
PROFESSOR
DEPARTMENT OF MEDICINE
SCHOOL OF MEDICINE
CHAO FAMILY COMPREHENSIVE CANCER CENTER
UNIVERSITY OF CALIFORNIA, IRVINE
IRVINE, CA 92697

LEGRAND, SARA H, PHD *
ASSOCIATE RESEARCH PROFESSOR
DUKE GLOBAL HEALTH INSTITUTE
DUKE UNIVERSITY
DURHAM, NC 27710

LIN, JENNY J., MD, MPH *
PROFESSOR
DIVISION OF GENERAL INTERNAL MEDICINE
DEPARTMENT OF MEDICINE
ICAHN SCHOOL OF MEDICINE AT MOUNT SINAI
NEW YORK, NY 10029

LYONS, ELIZABETH J, MPH, PHD
ASSOCIATE PROFESSOR
DEPARTMENT OF NUTRITION AND METABOLISM
THE UNIVERSITY OF TEXAS MEDICAL BRANCH
GALVESTON, TX 77550

MAZZEO, SUZANNE E, PHD *
PROFESSOR
DEPARTMENT OF PSYCHOLOGY
COLLEGE OF HUMANITIES AND SCIENCES
VIRGINIA COMMONWEALTH UNIVERSITY
RICHMOND, VA 23284

MILLER, BRENDA A, PHD *
SENIOR RESEARCH SCIENTIST
PREVENTION RESEARCH CENTER
PACIFIC INSTITUTE FOR RESEARCH AND EVALUATION
BERKELEY, CA 94704

MILLER, ELIZABETH, MD, PHD *
PROFESSOR OF PEDIATRICS
DEPARTMENT OF PEDIATRICS
UNIVERSITY OF PITTSBURGH SCHOOL OF MEDICINE
PITTSBURGH, PA 15213

NELSON, TOBEN FREDERICK, SCD
ASSOCIATE PROFESSOR
DIVISION OF EPIDEMIOLOGY
AND COMMUNITY HEALTH
SCHOOL OF PUBLIC HEALTH
UNIVERSITY OF MINNESOTA
MINNEAPOLIS, MN 55454

NUTOR, JERRY JOHN, PHD *
ASSISTANT PROFESSOR
DEPARTMENT OF FAMILY HEALTH CARE NURSING
UNIVERSITY OF CALIFORNIA SAN FRANCISCO
SAN FRANCISCO, CA 94143

PALACIOS, CRISTINA, PHD *
ASSOCIATE PROFESSOR
DEPARTMENT OF DIETETICS AND NUTRITION
ROBERT STEMPEL COLLEGE OF PUBLIC HEALTH
FLORIDA INTERNATIONAL UNIVERSITY
MIAMI, FL 33199

PARKER, ANDREA G, PHD *
ASSOCIATE PROFESSOR
SCHOOL OF INTERACTIVE COMPUTING
COLLEGE OF COMPUTER
GEORGIA INSTITUTE OF TECHNOLOGY
ATLANTA, GA 30308

PATEL, MINAL R, MPH, PHD *
ASSOCIATE PROFESSOR
DEPARTMENT OF HEALTH BEHAVIOR AND HEALTH
EDUCATION
SCHOOL OF PUBLIC HEALTH
UNIVERSITY OF MICHIGAN
ANN ARBOR, MI 48109

RISENDAL, BETSY C, PHD *
PROFESSOR
COMMUNITY AND BEHAVIORAL HEALTH
COLORADO SCHOOL OF PUBLIC HEALTH
UNIVERSITY OF COLORADO CANCER CENTER
AURORA, CO 80045

ROSAS, LISA GOLDMAN, MPH, PHD *
ASSISTANT PROFESSOR
DEPARTMENT OF EPIDEMIOLOGY AND POPULATION HEALTH
AND DEPARTMENT OF MEDICINE (DIVISION OF PRIMARY CARE AND POPULATION HEALTH)
STANFORD UNIVERSITY SCHOOL OF MEDICINE
PALO ALTO, CA 94305

SHAIBI, GABRIEL QUANTUM, PHD
PROFESSOR
CENTER FOR HEALTH PROMOTION AND DISEASE PREVENTION
COLLEGE OF NURSING & HEALTH INNOVATION
ARIZONA STATE UNIVERSITY
PHOENIX, AZ 85004

WANG, RUI, PHD
ASSOCIATE PROFESSOR
DEPARTMENT OF POPULATION MEDICINE
HARVARD PILGRIM HEALTH CARE INSTITUTE
HARVARD MEDICAL SCHOOL
BOSTON, MA 02215

WILLIAMS, LOVORIA B, PHD *
ASSOCIATE PROFESSOR
COLLEGE OF NURSING
UNIVERSITY OF KENTUCKY
LEXINGTON, KY 40536

ZHU, MOTAO, PHD, MD
PROFESSOR OF PEDIATRICS AND EPIDEMIOLOGY
CENTER FOR INJURY RESEARCH AND POLICY
DIVISION OF EPIDEMIOLOGY, COLLEGE OF PUBLIC HEALTH
DEPARTMENT OF PEDIATRICS, COLLEGE OF MEDICINE
THE OHIO STATE UNIVERSITY
COLUMBUS, OH 43205

SCIENTIFIC REVIEW OFFICER

DAGADU, HELENA ERYAM, MPH, PHD
SCIENTIFIC REVIEW OFFICER
CENTER FOR SCIENTIFIC REVIEW
NATIONAL INSTITUTE OF HEALTH
BETHESDA, MD 20892

EXTRAMURAL SUPPORT ASSISTANT

NJOKU, PHILIP C
EXTRAMURAL SUPPORT ASSISTANT
DIVISION OF AIDS, BEHAVORIAL, POPULATION SCIENCES
HEALTHCARE DELIVERY AND METHODOLOGIES (HDM)
NATIONAL INSTITUTES OF HEALTH
BETHESDA, MD 20892

* Temporary Member. For grant applications, temporary members may participate in the entire meeting or may review only selected applications as needed.

Consultants are required to absent themselves from the room during the review of any application if their presence would constitute or appear to constitute a conflict of interest.
